# Long-Tailed Silverfish (*Ctenolepisma longicaudata*) Control; Bait Choice Based on Primary and Secondary Poisoning

**DOI:** 10.3390/insects11030170

**Published:** 2020-03-07

**Authors:** Anders Aak, Morten Hage, Bjørn Arne Rukke

**Affiliations:** Norwegian Institute of Public Health, Department of Pest Control, Lovisenberggata 8, Postboks 4404 Nydalen, NO-0456 Oslo, Norway; anders.aak@fhi.no (A.A.); morten.hage@fhi.no (M.H.)

**Keywords:** durability, efficiency, IPM, pest, pesticide survival

## Abstract

The long-tailed silverfish (*Ctenolepisma longicaudata)* has recently made its appearance and demonstrated a tremendous proliferation in Norway, where it is currently considered a major indoor nuisance pest in modern buildings. To reduce the risk of human pesticide exposure, several baits with indoxacarb, clothianidin, fipronil or imidacloprid as the active ingredient were investigated to provide knowledge regarding their potential for integrated pest management solutions. Primary and secondary poisoning, as well as the durability of baits, were experimentally evaluated in bioassays. Baits with indoxacarb, clothianidin and fipronil killed more than 90% of the experimental insects (primary poisoning) when presented in competition with food. Only indoxacarb produced high mortality when dead conspecifics were consumed (secondary poisoning) and resulted in more than 75% mortality. The efficacy of baits with indoxacarb against *C. longicaudata* was also found to be very long. Laboratory degraded baits were consumed and induced high levels of mortality for more than a month, and field degraded baits for more than 6 months. Adults and juveniles were equally affected in the experiments. Primary and secondary toxicity in combination with long durability and effects on both life stages tested suggest that the bait has high-level potential as a safe control strategy against the long-tailed silverfish if indoxacarb is used as the active ingredient.

## 1. Introduction

The long-tailed silverfish (*Ctenolepisma longicaudata*, Zygentoma: Lepismatidae) has recently made its appearance and proliferated in several European countries [1,2,3,4,5,6,7,8,9,10]. It is considered a major indoor nuisance pest in Norway, where it typically appears in modern buildings [11]. The long-tailed silverfish cause minimal damage to objects in private homes [12,13,14], but they may generate stress and discomfort as they move around on floors, walls, cabinets and furniture throughout an infested locality [11]. They may feed on paper, pictures, old books or plant-based cloth [12,13], and for museums or other collection-oriented institutions it is a major concern because of the potential damage to rare and priceless items [15,16].

The long-tailed silverfish is considered more difficult to eradicate than other bristletail species such as the common silverfish (*Lepisma saccharina*) or the firebrat (*Thermobia domestica*) due to its ability to cope with drier conditions [17,18,19,20]. Aggregations typically appear close to food sources with suitable environmental conditions [12,13]. They utilize a wide variety of foods [17], and although an indoor environment may be considered clean, there may still be adequate food sources for population growth. They are known to feed on leftovers such as sugars, proteins and fat [12,13,14,17]. This may allow the use of baits in line with the successful strategies applied against cockroaches [21,22]. Previous investigations of the effect of baits against the common silverfish and the firebrat have shown that baits were able to induce significant mortality, and some of the active ingredients did not appear to affect the amounts ingested [23].

All baits tested in this study have active ingredients that are neurotoxic to insects. Indoxacarb blocks neuronal sodium channels [24], neonicotinoids like imidacloprid and clothianidin act on post-synaptic nicotinic receptors where they bind to nicotinic acetylcholine receptors [25,26], while fipronil blocks GABA receptors in the nervous system [25].

Currently, there is limited scientific knowledge about baits and their active ingredients to combat the long-tailed silverfish, but anecdotal claims and unquantified field experience suggests that bait strategies have limited effectiveness [12]. If, however, the available modern baits are sufficiently attractive or nutritionally stimulating enough to encourage feeding, a potential tool against this new European nuisance pest may become available. A bait strategy is particularly favourable because of the low health risk they hold compared to conventional application of spray insecticides [21,27]. Baits may also be durable when applied in secluded spots and in cockroach control, as they induce primary poisoning through ingestion, secondary poisoning through excretions, necrophagy or emetophagy and tertiary poisoning through the same mechanisms, but are delivered by secondary donors [28]. These aspects of extended effects from baits point in favour of potentially successful long-tailed silverfish control. Its lifecycle resembles the cockroach lifecycle with a slow progression, many juvenile stages and multiple feeding events before reproduction and a significant time spent in aggregations where secondary poisoning may have an impact.

In this study we investigate the effect of several commercially available baits with a potential to be used against the long-tailed silverfish. Primary and secondary poisoning are evaluated as well as the durability of the bait, and the results contribute new knowledge regarding long-tailed silverfish control.

## 2. Materials and Methods

Individuals (n > 40) of *C. longicaudata* were collected from each of four localities in and around Oslo city in 2016 and 2017. These insects were used to establish breeding colonies under laboratory conditions. Colonies are maintained in plastic terrariums with paper on the floor, various available hiding places and a continuous supply of goldfish flake (Tetra GmbH, Melle, Germany) and ecologic flaked oat for feeding (Axa Økologiske havregryn, Oslo, Norway). They have constant access to moisture through glass tubes filled with water and closed with a cotton wick, and dry cotton is used for deposition of eggs. The rearing room maintained a temperature of 23–25 °C, a relative humidity of 60% and a 16:8 h-light/dark cycle.

**Figure 1:**
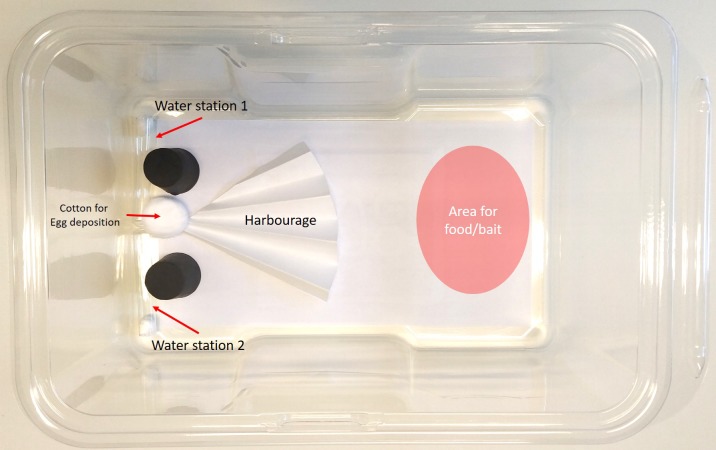
Arenas for bait testing with *Ctenolepisma longicaudata.*

The arenas used for bait testing (Figure 1) were constructed from disposable mouse cages (Innocage mouse, San Diego, CA, USA) with a 14.5 cm × 27 cm premium label paper (Herma GmbH, Filderstad, Germany) adhered to the floor of the cage. In one end of the arena we placed two glass tube water stations held in place by rubber stoppers and one dry cotton ball (0.5 g, Cutisoft, Hamburg, Germany) for egg deposition. In the centre of the arena, but offset to the same side as the water stations, we used ¼ of a pre-folded filter paper (Whatman 2555 ½, Germany Healthcare, Buckinghamshire, UK) to create hiding places for the experimental insects. The opposite half of the arena was used to present the food/bait according to the needs of the experiment.

Experimental insects were moved from the stock cultures to experimental arenas by letting them crawl onto a filter paper and lifting them into the test arena. After introduction to the arena, all the experimental insects were starved (only water in the arena) for three days before introduction of food/bait. Mortality was checked daily by observation of self-motions or, if experimental insects appeared moribund, by touching them gently with a fine hairbrush. Experiments were conducted in Sanyo climate chambers (MLR-351H, Medinor ASA, Oslo, Norway) with the same conditions as the rearing room, and all results were analysed in JMP^®^ Pro 14.1.0 (SAS institute, Cary, NC, USA) using Kaplan-Meier survival analyses. To identify significant differences between groups we used the log-rank test with the *p*-Value limit set to *p* < 0.05.

**Experiment** **1.** *Screening for suitable bait candidates:*

**Figure 2:**
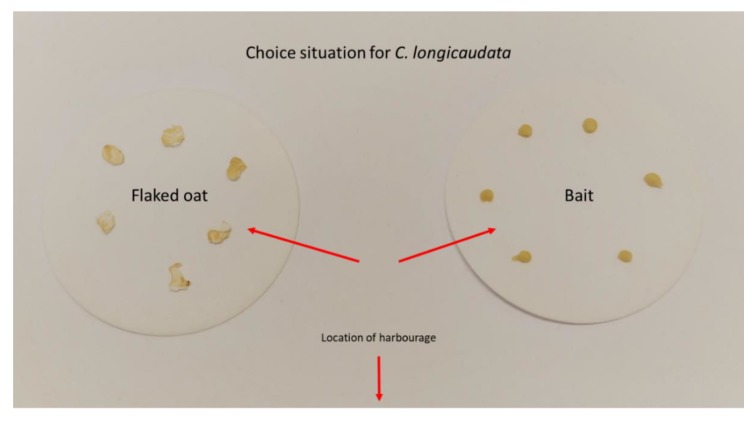
Set-up for choice between food and bait when evaluating commercially available baits against *Ctenolepisma longicaudata* in arena bioassays.

We used four arenas per experimental treatment, and each arena held two male and two female adults in combination with four juveniles (individuals with scales, but not larger than 7 mm long). This provided a total of 32 individuals per bait tested. Baits were tested in parallel with flaked oat grains to create a resemblance of the natural conditions in private homes where small amounts of dry food will often be accessible on the floor. To provide the long-tailed silverfish with a choice situation, we placed six flaked oat grains on one side of the feeding area and six droplets of baits of the same size as the oat grains on the other (average weight (±SE); oat grains = 5.24 mg (±0.32) and bait droplets = 7.99 mg ± (0.46), Figure 2). The control group received only flaked oat grain. Baits were selected according to availability for the Norwegian pest control industry, and we made sure there was variation in the different active components. Only gel/paste-formulations were tested. Four baits were intended for cockroaches (Maxforce white IC—active ingredient: 2.15% imidacloprid (Bayer, Leverkusen, Germany), Maxforce Platin—active ingredient: 0.01% clothianidin (Bayer, Leverkusen, Germany), Goliath gel—active ingredient: 0.05% fipronil (BASF, Ludwigshafen, Germany) and Advion cockroach—active ingredient: 0.6% indoxacarb (Syngenta, Basel, Switzerland)), and two baits were intended for ants (Maxforce quantum—active ingredient: 0.03% imidacloprid (Bayer, Leverkusen, Germany) and Advion ant—active ingredient: 0.05% indoxacarb (Syngenta, Basel, Switzerland)). Dead insects were removed daily, and the experiment was terminated on day 18 after introduction of the food/bait.

**Experiment** **2.** *Secondary poisoning:*

Based on results from experiment 1, insects that had died from ingestion of the two indoxacarb or the clothianidin treatments were used for investigation of secondary poisoning. The insects, having died from primary ingestion (29 to 31 individuals per experiment) of the three products, were kept separate and stored in a freezer before being placed in the test arena in this experiment. The dead insects were equally divided between four arenas with six adults and six juvenile individuals to provide a total of 48 insects per bait. No competing food source was present in this experiment. Because it was difficult to differentiate between insects that had died previously and those dying from secondary poisoning, dead individuals were not removed. The experiment was terminated on day 18 after the introduction of dead specimens. Bait with fipronil was not investigated for secondary poisoning because of its greater toxicity for humans, and due to its UV-light conversion to fipronil-desulfinyl, which further increases toxicity [29,30,31].

**Experiment** **3.** *Durability of bait in the laboratory:*

Based on results from the secondary poisoning experiment, baits with indoxacarb were tested for efficiency after having been dried for two or four weeks. This was done to investigate if bait palatability and active ingredient efficacy was affected when the gel becomes older and develops a surface crust. The bait drops were kept at 22 °C and 60%RH for the required time and introduced into the arenas to create an exact replicate of experiment 1 in all respects except age of the bait.

**Experiment** **4.** *Durability of bait in the field:*

Baits with indoxacarb have been applied in several field experiments in Norway during the past two years. Twenty-four small bait drops (0.05% and 0.6% indoxacarb) were collected after four and six months to check if their efficacy after field degradation was reduced. The field-collected bait drops were introduced into the arenas as for experiment 1 but presented without competing food sources to more distinctly identify sustained function. We used four arenas per experimental treatment, and each arena held two male and two female adults in combination with four juveniles for a total of 32 individuals per bait tested. Each arena received two small field-collected bait drops (8.00 ± 1.0 mg per arena).

## 3. Results

**Experiment** **1.** *Screening for suitable bait candidates:*

**Figure 3:**
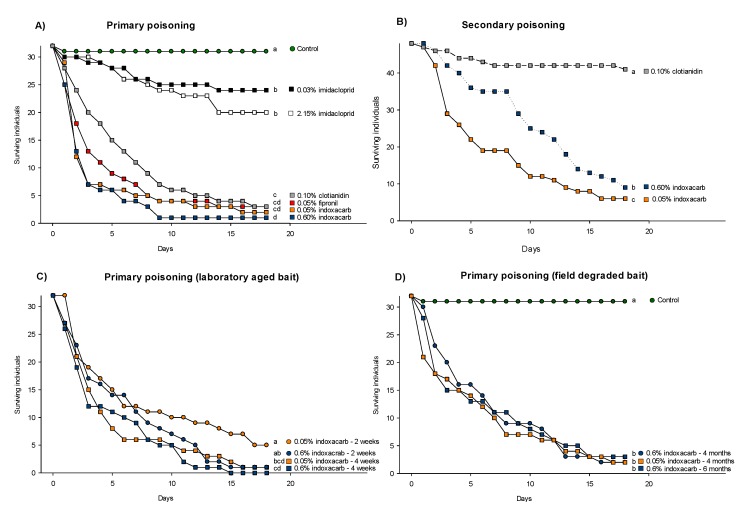
Survival of *Ctenolepisma longicaudata* in bio-assays with (**A**) five commercial baits presented in competition with a commonly preferred food source (flaked oat grains), (**B**) access to conspecifics having died from ingestion of three different baits, (**C**) two commercial baits having been aged for two or four weeks before being presented in competition with a commonly preferred food source (flaked oat grains) and (**D**) two commercial baits having been aged four or six months under field conditions before being placed in the arena as the only food source available. The different letters (a, b, c, and d) indicate significant differences in survival between pairs of treatments (Kaplan-Meier survival analysis, the log-rank test with the *p*-Value limit set *p* < 0.05). The commercially available baits that were tested are: (white) Maxforce white IC, (gray) Maxforce Platin, (red) Goliath gel, (blue) Advion cockroach (black) Maxforce quantum and (yellow) Advion ant. The control treatment is assigned a green colour.

All baits significantly reduced survival relative to the control (Kaplan-Meier log rank test – only least significant test shown; imidacloprid 0.03% vs. Control: χ^2^ = 6.10, *p* = 0.014, Figure 3A). Adults and juveniles died in equal numbers across all treatments, and the mean adult–juvenile mortality ratio was 51.0% to 49.0% (±1.3 SE). Baits with fipronil, clothianidin and indoxacarb caused significantly higher mortality compared to imidacloprid, which did not even reach 50% mortality (Kaplan-Meier log rank test – only least significant test shown; 2.15% imidacloprid vs. 0.1% clothianidin: χ^2^ = 24.78, *p* < 0.001, Figure 3A). Among the four baits that killed between 91% and 97% of the insects, there was a significant difference between only 0.6% indoxacarb and 0.1% clothianidin (Kaplan-Meier log rank test; χ^2^ = 7.47, *p* = 0.006, Figure 3A).

**Experiment** **2.** *Secondary poisoning:*

Survival was strongly reduced when the long-tailed silverfish fed on carcasses killed by indoxacarb. More than 75% secondary mortality was observed compared to the clothianidin treatment which resulted in 15% mortality. All three treatments were significantly different from each other, and the lowest dose of 0.05% indoxacarb resulted in the highest secondary mortality (Kaplan-Meier log rank test; 0.1% clothianidin vs. 0.6% indoxacarb: χ^2^ = 44.24, *p* < 0.001 and 0.6% indoxacarb vs. 0.05% indoxacarb: χ^2^ = 5.69, *p* = 0.017, Figure 3B).

**Experiment** **3.** *Durability of bait in the laboratory:*

The laboratory-aged bait appeared comparable to the fresh bait as the bulk of the mortality (more than 50%) occurred within the first week. However, both four-week-old baits resulted in significantly higher mortality compared to their respective two-week-old baits (Kaplan-Meier log rank test; two-week-old vs. four-week-old 0.05% indoxacarb: χ^2^ = 4.52, *p* = 0.034, and two-week-old vs. four-week-old 0.6% indoxacarb: χ^2^ = 4.51, *p* = 0.034, Figure 3C). These differences were mostly due to time of death because all baits resulted in five or less survivors after 18 days.

**Experiment** **4.** *Durability of bait in the field:*

Field-collected four- and six-month old baits with indoxacarb maintained their ability to significantly reduce survival compared to the control (Kaplan-Meier log rank test – only least significant test shown; Control vs. four-month old 0.6% indoxacarb bait: χ^2^ = 52.86, *p* < 0.001, Figure 3D). There was no difference between the two four-month-old baits (Kaplan-Meier log rank test; 0.05% indoxacarb vs. 0.6% indoxacarb: χ^2^ = 0.34, *p* = 0.561, Figure 3D) or between the four- and six-month-old 0.6% indoxacarb baits (Kaplan-Meier log rank test; χ^2^ = 0.10, *p* = 0.751, Figure 3D).

## 4. Discussion

This study provides knowledge regarding four important aspects of bait as a strategy against the long-tailed silverfish. Firstly, products with indoxacarb, clothianidin and fipronil kill long-tailed silverfish in competition with other food sources. Secondly, among the functional baits, indoxacarb is preferable as an active ingredient when compared to clothianidin because of its ability to induce much higher levels of secondary poisoning. Thirdly, baits kill both adults and juveniles and may therefore target individuals both before and during reproduction. Finally, we have shown that field durability of the killing agents is very long, and that long-tailed silverfish encountering bait droplets four to six months after application eat the bait and die. Altogether, these findings suggest that bait has a high potential as a tool against the long-tailed silverfish when the proper active ingredient is used.

Hardly anything is known about the natural biology of the long-tailed-silverfish, and its food preferences are only described from laboratory or indoor conditions [12,13,14,17]. Silverfish in general are often described as paper-eating insects, although there are distinct differences between species in their enzymatic ability to handle cellulose [32], and the paper utilization depends on complex and species-specific interactions with bacteria and fungi affecting both digestion and aggregation formation [33,34,35,36,37]. Environmental conditions may also adjust the food preference in bristletails [38]. The long-tailed silverfish, however, is described as having an autonomous feeding response when encountering sugars [17], and when supplied with alternate food such as insects, conspecifics and grain under laboratory conditions, they hardly consume paper as nutrition (personal observation). These feeding habits indicate a complex diet in the long-tailed silverfish, as opposed to that of a paper-feeding insect, and suggest it is an opportunistic feeder on the most available food sources. Insects, including bristletails, seek sugar as an energy source; proteins are in demand for production of eggs or growth, and fat is used for energy and hormone production [33,34,38,39]. The long-tailed silverfish’s broad diet is, therefore, expected and concurs with the observed mortality in our bioassays, where we found high primary mortality from nutrient-rich baits and a strong secondary mortality from ingestion of protein-rich conspecifics. The carrier gels in the baits are trade secrets, but most of the formulations are likely to contain mixtures of sugars, proteins and lipids [21,22,40]. Even though the baits used in this study are optimized for species other than silverfish, the most efficient ones were able to kill most individuals in our experimental populations in competition with another available food source. The few survivors may be foraging individuals that may, by chance, have encountered flaked oats only, or passive individuals having entered the non-feeding final third of its current instar during which they simply await the moulting process [17]. On the final day of testing, we observed that, compared to the control animals, most surviving experimental insects showed abnormal escape behaviour when touched with a hairbrush. This indicates a negative impact from the pesticides even though mortality did not occur within 18 days. Sublethal effects from toxins are known from many insect groups [41,42,43], and the appearance of irregular behaviour might be an indication of feeding aversion or fast acting toxins causing neurological disorders to disrupt the ingestion before a lethal dose was obtained. The production of weakened or dysfunctional individuals, however, is likely to increase the negative long-term effect on pest populations.

The secondary poisoning that yielded up to 90% mortality is remarkable and, as we did not remove the dead individuals, tertiary poisoning may also have occurred. Intense feeding on conspecifics is likely explained by a need for proteins or by acquisition of gut-symbionts [33,34,35], and the level of secondary poisoning points to dead conspecifics as an important part of the diet. This may be beneficial for field application of baits because good sources of proteins are often in limited supply for indoor insects and may force long-tailed silverfish populations to rely heavily on dead conspecifics. Protein sources such as other dead insects and human food leftovers can easily be kept at a minimum during control. The secondary poisoning also appears to be dependent on the primary toxicity of the different baits. Indoxacarb needs to be ingested and bioconverted before killing the insect [44,45], whereas clothianidin acts more instantly. The latter may consequently provide an insufficient accumulation of toxins in the dead individual to induce efficient horizontal transfer [21,28,46]. The secondary poisoning experiment showed that the least toxic bait induced the highest levels of secondary mortality, and may therefore mirror the amount ingested before death. This suggests that a low dose of a slower acting pesticide may actually be better to control silverfish. The very low indoxacarb dose in the ant bait (1/12 of the cockroach bait) was sufficient to kill the primary target and appears to have produced more potent carcasses for secondary poisoning. Indoxacarb in bait formulations has also been shown to leave limited amounts of residues in cockroach-infested dwellings [27], and even lower doses in the ant bait are able to assure a very safe approach. It is also worth noting that four-week-old baits resulted in slightly higher primary mortality compared to two-week-old baits. This might be an effect of drying of the bait to give slightly higher concentrations of the active ingredients. Considerations regarding primary, secondary and a potential tertiary poisoning against concentration of the active ingredient require further studies.

It is also interesting that the baits tested here appear to be more efficient than those previously tested against the common silverfish and the firebrat [23]. Whether this is a result of the bioassay design, active ingredients, palatability of carrier gels or species-specific foraging habits is not known. The observed primary and secondary effects, however, warrant further experiments with indoxacarb against other urban bristletails. Species-specific food preference may then also be integrated for the enhancement of control efficiency. However, the rapid and persistent mortality occurring within one week in this study indicate that the four best baits are capable of having a major impact on long-tailed silverfish populations even without the secondary poisoning. The long duration of the baits and the willingness to feed on fresh, semi-dry (two to four weeks) and dry (six months) bait also indicate that the long-tailed silverfish may consume the bait for an extended time. While cockroaches reduce their feeding on drier baits [47] and may require reapplication for proper field effect, the long-tailed silverfish appear to offer the benefit of a low intensity application strategy in an IPM program. Removal of competing food sources before and after application benefits bait strategies against cockroaches [22,48] and should also be done in silverfish control.

Compared with most other types of pesticides, baits limit technicians’ and residents’ risk of exposure to harmful substances and should consequently be the method of choice against the long-tailed silverfish when control efforts are deemed necessary [21,22,27,49]. Traditional spray application may also require treatment in many rooms as the long-tailed silverfish disperse to many parts of the building or the infested dwelling [11]. It is also assumed that they spend most of their time in hidden locations such as wall voids, behind skirtings, underneath floorboards, inside furniture shelfs or other partially enclosed parts of the building [12,13]. These places are out of reach from spray application unless labour intensive methods are used to remove the objects that create the hiding places. Due to their elusive and cryptic behaviour, the long-tailed silverfish may linger in the constructions for a long time, and it may be more rational to let them freely search out the bait and possibly find their way back to directly target conspecifics in aggregations through secondary poisoning.

## 5. Conclusions

Based on this study it appears likely that the long-tailed silverfish populations may be decimated with a success comparable to cockroach control [49,50,51,52,53,54,55] by use of baits with indoxacarb. Handbooks in pest control often point to baits as a strategy with limited effect against some bristletails [12,13], but this study might contribute towards the re-evaluation of this notion if field efficiency is also investigated and documented.
